# On the (mis)calculation of forensic science error rates

**DOI:** 10.1073/pnas.2215695119

**Published:** 2022-12-19

**Authors:** Jeff Kukucka

**Affiliations:** ^a^Department of Psychology, Towson University, Towson, MD 21252

Hicklin et al. (1) recently assessed the accuracy of handwriting comparisons among professional forensic document examiners. Their study has many strengths. However, I have serious concerns over how they calculated examiners’ false positive error rate—i.e., how often examiners made incorrect judgments that wrongly implicate an innocent person.

In their study, 86 examiners collectively made 3,713 decisions about nonmated handwriting sets (i.e., samples written by different individuals). Of these, examiners misjudged nonmated sets as written by the same person 114 times, hence the purported false positive rate of 3.1% (114/3,713).

First, despite characterizing inconclusive decisions as “neither correct nor incorrect,” the authors effectively counted them as correct by including them in their error rate calculations. When assessing the accuracy of examiners’ *judgments*, inconclusive decisions should be excluded from the error rate denominator (2, 3), which increases the false positive rate to 3.6% (114/3,166).

Second, examiners judged each set as either definitely, probably, probably not, or definitely not written by the same person (or inconclusive). Critically, the authors defined “errors” as judgments that were both incorrect *and definitive*, arguing that “in practice… definitive and qualified conclusions [are] intended to convey different strengths.” However, definitive and qualified (e.g., “more likely than not”) conclusions have an equivalent impact on juror decision-making (4, 5) and are thus equally harmful in practice when incorrect. It therefore seems appropriate to include both “definite” and “probable” judgments in the error rate numerator, which raises the false positive rate to 8.2% [(114 + 147)/3,166].

Third, many examiners simply declined to compare some of the assigned sets—a luxury they do not have in actual casework. If we consider only the examiners who compared *all* of the assigned sets (see Table S27), after excluding inconclusives and including qualified judgments as described above, the false positive rate increases to 9.1% (217/2,388).

To summarize, examiners in Hicklin et al. who compared all sets under relatively “ideal” conditions (e.g., suitable materials, minimal pressure; 6, 7) wrongly implicated an innocent person in one out of every 11 judgments. This is alarming given that the examiners in this study also reported having collectively “testified as an expert… in a legal setting” approximately *one thousand* times.

Although the authors use reproducibility data to argue that verification would detect many such errors, their methodology assumes universal and blind verification, which is ideal but rare in practice (8, 9). Moreover, false positive errors (whether definitive or qualified) were not limited to a subset of samples or examiners; they occurred for 87% of nonmated sets (89/102) and among 89% of examiners who judged at least 40 nonmated sets (57/64).

As per Hicklin et al., their “study was conducted to provide data… for use by policymakers, laboratory managers, the legal community, and forensic document examiners.” While impressive, I fear that its purported error rate could dangerously mislead stakeholders if presented unchallenged. Moreover, these authors have since published a study of footwear comparison (10) that raises some of these same concerns. As we work to strengthen forensic science, we must communicate our findings to nonscientists with appropriate nuance.
